# Underrecognized Thrombotic Risk in Klinefelter Syndrome: A Clinical Insight

**DOI:** 10.7759/cureus.102884

**Published:** 2026-02-03

**Authors:** Hrithik Dakssesh Putta Nagarajan, Mohammed Afsharhussain Hithayathulla, Tejashvi Rameshkumar, Md Ramij Biswas, Nitish Thirugnanasambandam

**Affiliations:** 1 Department of Internal Medicine, Madurai Medical College, Madurai, IND; 2 Department of Internal Medicine, K.A.P. Viswanatham Government Medical College, Tiruchirappalli, IND; 3 Department of Internal Medicine, Rajshree Medical Research Institute, Bareilly, IND; 4 Department of Internal Medicine, Dr RK Diabetic Foot and Podiatry Institute, Chennai, IND

**Keywords:** case report, clinical case report, deep vein thrombosis (dvt), klinefelter, thrombophilia

## Abstract

Klinefelter syndrome (KS), the most common male aneuploidy (47,XXY), is linked to hypogonadism, gynecomastia, and infertility, with an established but underrecognized association with venous thromboembolism and limited data on mechanisms in patients without additional thrombophilic conditions. We report the case of a 33-year-old South Asian male with subacute-to-chronic deep vein thrombosis (DVT) of the right leg and absent traditional thrombosis risk factors. Clinical evaluation, hormonal assays, and karyotyping confirmed KS. Extensive investigations ruled out congenital and acquired thrombophilias. The patient responded well to anticoagulation therapy, achieving symptom resolution. This case highlights the need for awareness of thrombotic risks in KS and underscores the importance of further research into the underlying mechanisms of this association.

## Introduction

Klinefelter syndrome (KS), named after Dr. Harry Klinefelter in 1942, occurs when a phenotypic male has two or more X chromosomes [1]. The most common genotype is 47,XXY, in more than 90% of the cases [2].

Klinefelter syndrome is the most common form of aneuploidy and is known to affect around 1:500 to 1:1000 males [3]. Multiple cohort and mechanistic studies have demonstrated a significantly increased risk of venous thromboembolism in patients with Klinefelter syndrome; however, data remain limited regarding underlying mechanisms and risk in patients without coexisting thrombophilic conditions. Several studies have demonstrated that patients with Klinefelter syndrome have a four- to eightfold increased risk of thrombosis compared with the general population, a risk comparable to that observed in individuals with inherited thrombophilias [4].

In this case report, we describe an intriguing presentation of Klinefelter syndrome in a 33-year-old South Asian male who presented with clinical features suggestive of subacute to chronic deep vein thrombosis (DVT) of the right lower limb. Through this case, we aim to emphasize the increased risk of venous thromboembolism in patients with Klinefelter syndrome, particularly in the absence of identifiable inherited or acquired thrombophilic factors, a subgroup that remains underrepresented in the existing literature. Heightened awareness of this association may enable earlier recognition and optimized management of thromboembolic complications occurring during the course of the syndrome.

## Case presentation

Case history and examination

A 33-year-old South Asian male came to the clinic with complaints of swelling and pain in the right leg for the past month, raising suspicion of lower limb venous thrombosis. The patient did not have any history of factors that are typically known for causing venous thrombosis, vis-à-vis no history of trauma and no history of recent surgery or immobilization. The patient did not report any loss of weight or loss of appetite.

On general examination, there was no cyanosis, anemia, clubbing, or lymphadenopathy, and the vitals were within normal range. The body mass index (BMI) was also normal. On local examination, there was swelling of the right lower limb from the foot up to the thighs. On further examination, sparse upper lip and axillary hair were noted, and bilateral gynecomastia was also noted. Both testes were palpable but were smaller in size, with the long diameter measuring around 3 cm. Examination of the systems was normal.

Investigations

**Figure 1:**
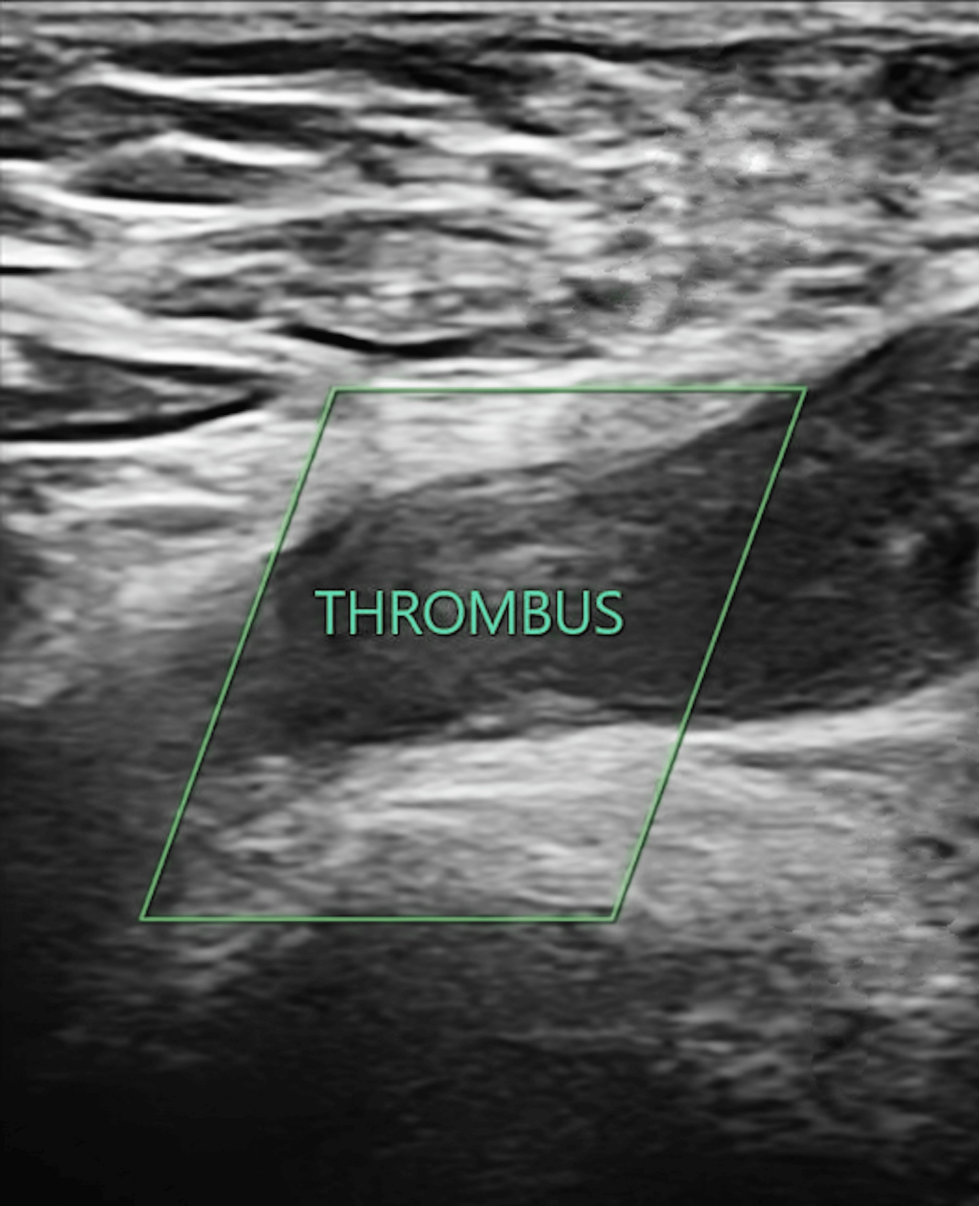
Ultrasonographic evidence of deep vein thrombosis (DVT) in the right common femoral vein. Grayscale compression ultrasonography of the right common femoral vein demonstrating an intraluminal echogenic thrombus (outlined), consistent with subacute-to-chronic deep vein thrombosis.

Initial laboratory studies, including coagulation studies, were within normal range (Table 1). On venous Doppler, thrombi were found in the right common femoral vein (Figure 1) and superficial and deep femoral veins, confirming the diagnosis of lower limb DVT. An ultrasound of the abdomen was done to rule out the possibility of malignant causes of hypercoagulability, but the results were normal.

**Table 1:** Baseline laboratory investigations. Initial laboratory investigations, including coagulation studies, were largely within normal limits, with no evidence of an underlying coagulation abnormality contributing to the thrombotic event. Mild anemia and thrombocytopenia were noted and were deemed incidental, with no specific cause identified. Values are presented as absolute numbers, and reference ranges correspond to standard adult laboratory values and may vary between laboratories.

Category	Parameter	Result	Reference Range
Hematology	Total Leukocyte Count (TLC)	9,900 /µL	4,000 – 11,000 /µL
	Hemoglobin (Hb)	10 g/dL	♂ 13–17 / ♀ 12–15 g/dL
	Packed Cell Volume (PCV)	29.8 %	♂ 40–50 % / ♀ 36–46 %
	Platelet Count (PLT)	1,26,000 /µL	1,50,000 – 4,50,000 /µL
	Mean Corpuscular Volume (MCV)	83 fL	80 – 100 fL
	Neutrophils	36%	40 – 70%
	Lymphocytes	43%	20 – 40%
	Monocytes	20%	2 – 10%
	Erythrocyte Sedimentation Rate (ESR)	42 mm/hr	♂ <15 / ♀ <20 mm/hr
Renal Function	Urea	20 mg/dL	15 – 40 mg/dL
	Creatinine	1.0 mg/dL	0.6 – 1.2 mg/dL
Electrolytes	Sodium (Na⁺)	134 mmol/L	135 – 145 mmol/L
	Potassium (K⁺)	3.6 mmol/L	3.5 – 5.0 mmol/L
Liver Function	Total Bilirubin	1.0 mg/dL	0.2 – 1.2 mg/dL
	Direct Bilirubin	0.7 mg/dL	<0.3 mg/dL
	Indirect Bilirubin	0.3 mg/dL	0.2 – 0.8 mg/dL
	SGOT (Aspartate Aminotransferase)	18 U/L	<40 U/L
	SGPT (Alanine Aminotransferase)	15 U/L	<40 U/L
	Alkaline Phosphatase (ALP)	84 U/L	40 – 130 U/L
	Serum Total Protein	7.6 g/dL	6.0 – 8.0 g/dL
	Serum Albumin	4.1 g/dL	3.5 – 5.0 g/dL
	Serum Globulin	3.5 g/dL	2.0 – 3.5 g/dL
Coagulation Profile	Prothrombin Time (PT)	14.4 sec	11 – 15 sec
	International Normalized Ratio (INR)	1.1	0.8 – 1.2
	Activated Partial Thromboplastin Time (aPTT)	32.5 sec	25 – 35 sec

**Figure 2:**
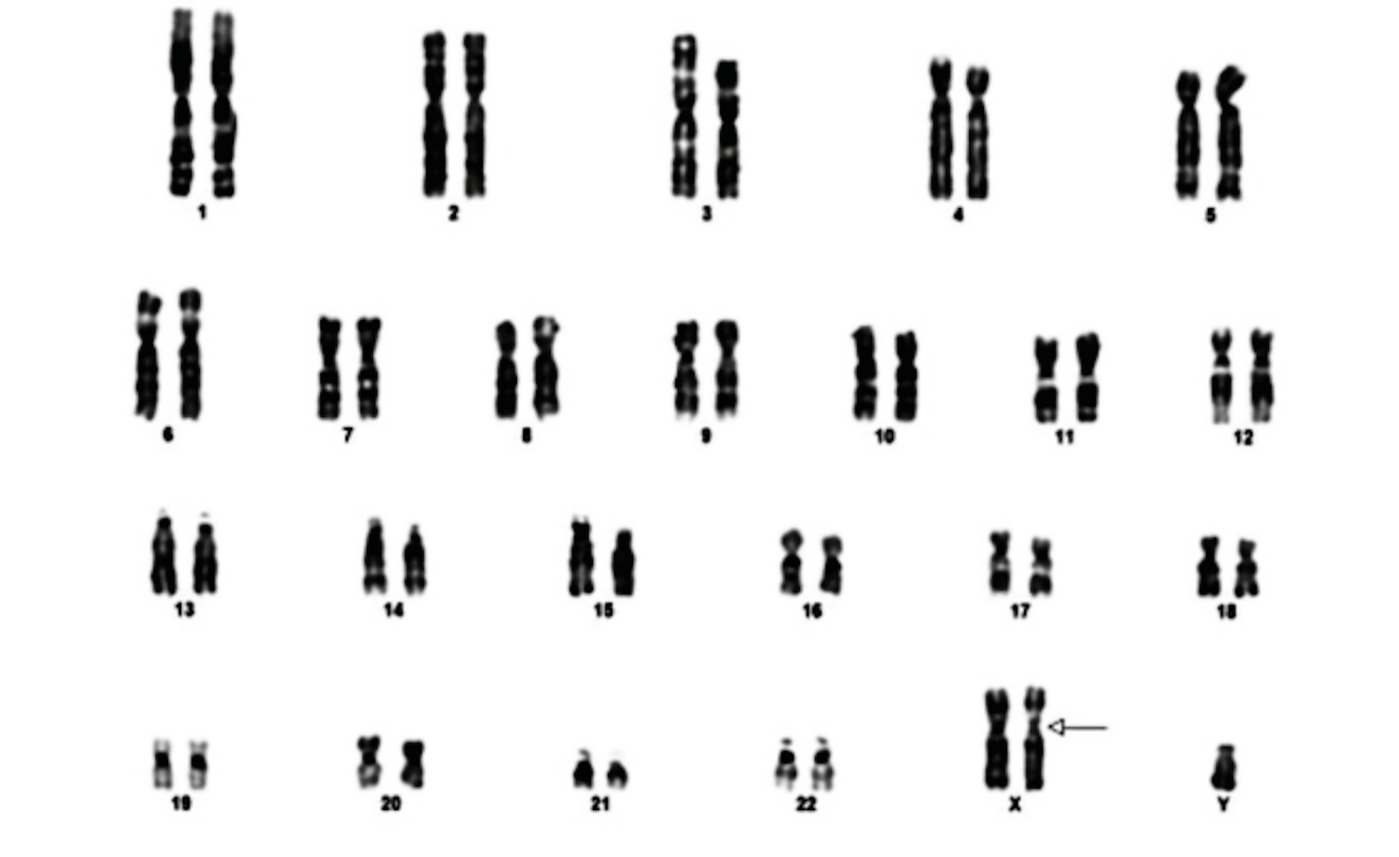
Karyotype analysis of the patient. The analysis depicts an extra X chromosome (marked with an arrow), confirming the genotype of the patient 47,XXY, and thereby confirming the diagnosis of Klinefelter syndrome.

The presence of hypogonadism and gynecomastia required further evaluation. So a buccal smear study was done. The smear showed the presence of Barr bodies, and karyotyping (Figure 2) and hormonal assays (Table 2) were suggested for further evaluation.

**Table 2:** Hormonal assay study of the patient. Markedly elevated gonadotropin levels with low serum testosterone were consistent with primary hypogonadism. However, the thyroid hormone values were within the normal range.

Hormone	Patient values	Reference ranges (for males)
Follicle-stimulating hormone (FSH)	52.06 mIU/ml	1.7 – 8.6 mIU/ml
Luteinizing hormone (LH)	45.89 mIU/ml	1.5 – 12.4 mIU/ml
Prolactin	10.93 ng/ml	4.74 – 23.3 ng/ml
Testosterone	107.8 ng/dl	249 – 836 ng/dl
Thyroid-stimulating hormone (TSH)	2.12 µIU/ml	0.3 – 5.5 µIU/ml
Free thyroxine (FT_4_)	1.26 ng/dl	0.89 – 1.72 ng/dl

The hormonal assay results and karyotype analysis were strongly supportive of a diagnosis of Klinefelter syndrome. To further investigate the cause of deep vein thrombosis, further studies, including tests for systemic lupus erythematosus (antinuclear antibody) and antiphospholipid antibody syndrome (lupus anticoagulant, anti-β2 microglobulin antibody, and anti-cardiolipin antibody), were done. The results of these tests came back negative. To rule out the probability of congenital causes of hypercoagulability, real-time polymerase chain reaction tests for the presence of factor V Leiden mutation (R506Q), factor II prothrombin gene mutation (G20210A), and methylenetetrahydrofolate reductase gene mutation (C677T) were done, but the results of all these tests came back negative. 

Treatment and follow-up

The patient's lower limb DVT was treated with injection heparin 5000 IU QID, a right lower limb compression bandage, and elevation. Although current guidelines recommend therapeutic weight-based low-molecular-weight heparin or continuous intravenous unfractionated heparin infusion for established deep vein thrombosis, unfractionated heparin was used in this case based on institutional practice and feasibility considerations. Tablet paracetamol 500 mg was prescribed for the associated pain, as and when needed. The patient's symptoms gradually subsided, and he was educated about his diagnosis of KS and its significant association with thrombophilia.

The patient was discharged on tablet nicoumarol 2 mg for sustained anticoagulation effects. Nicoumarol was selected for long-term anticoagulation due to local availability, cost considerations, and the feasibility of international normalized ratio (INR) monitoring. Given the unprovoked nature of the event and the ongoing hypercoagulable risk associated with Klinefelter syndrome, extended-duration anticoagulation was planned, with periodic reassessment of bleeding risk with a target INR of 2.0 - 3.0. On follow-up one month after discharge, the patient did not have any residual symptoms, and the INR was within the target therapeutic range.

## Discussion

**Figure 3:**
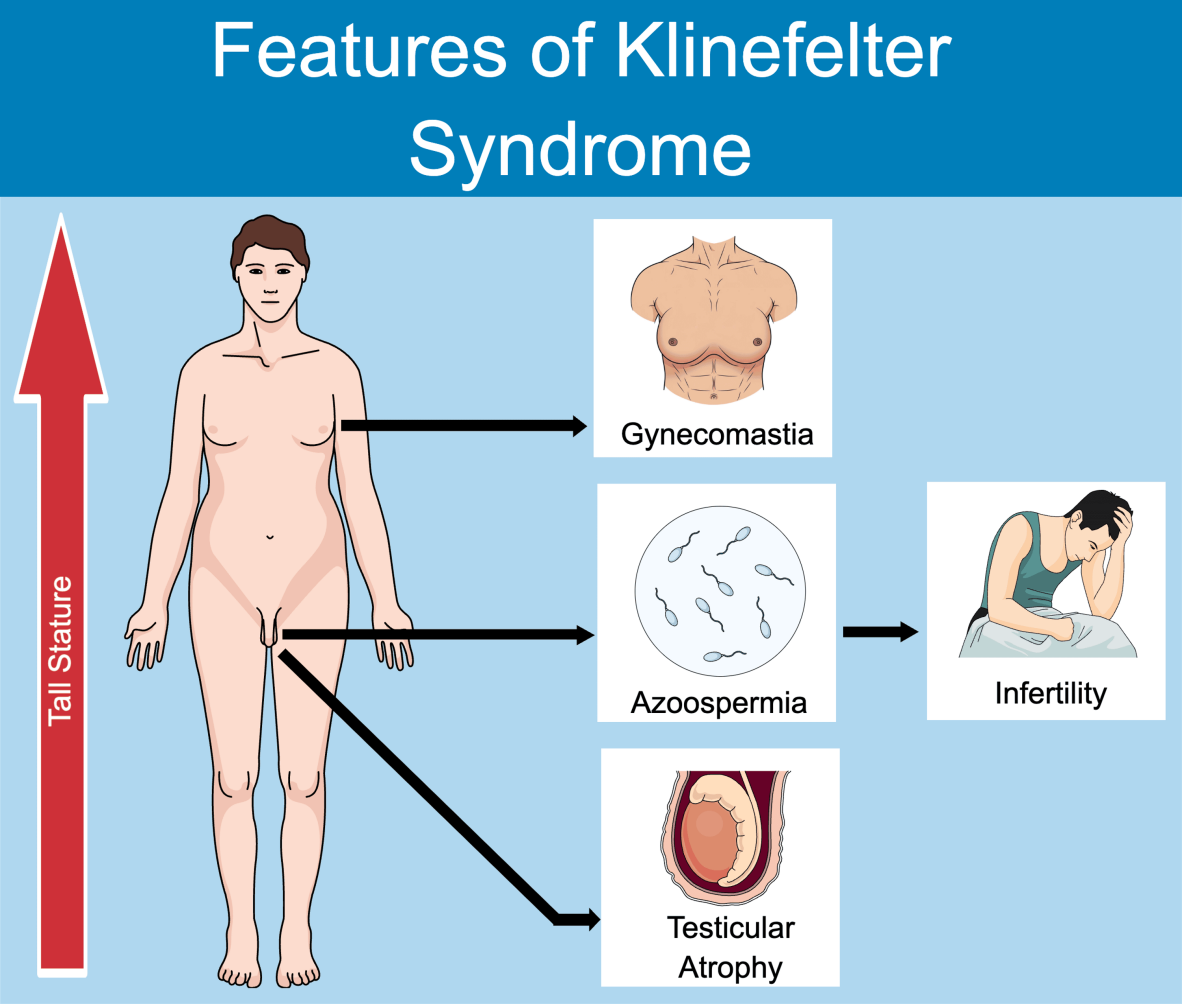
Simple graphical representation of the common features of males affected by Klinefelter syndrome. Image created by the authors based on Bonomi et al. [1].

The most common features of males affected with Klinefelter syndrome (KS) include a tall stature, gynecomastia, testicular atrophy, and azoospermia leading to infertility (Figure 3). But this syndrome is neither inherited nor inheritable [1].

Reported cases of venous thromboembolism in Klinefelter syndrome include those with and without coexisting hereditary thrombophilia. The present case belongs to the latter group, suggesting an intrinsic prothrombotic association with Klinefelter syndrome. Some of these articles suggest that the risk of thrombophilia in patients with KS is similar to that of patients with inherited thrombophilias, such as factor V Leiden and the prothrombin G20210A mutations [5]. Also, the overall prevalence of KS is similar to that of deficiencies of antithrombin, protein C, and protein S [4,5]. There is evidence of hypercoagulability in patients with KS before the initiation of treatment for KS in the medical literature. This effectively rules out the probability of the treatment of KS, such as testosterone, being the cause of increased coagulability in patients with KS [4].

The mechanism behind this association is unclear, but it is thought to be multifactorial. The increased dosage of the X-linked genes is one of the popular hypotheses. The gene for coagulation factor VIII is coded in the X chromosome, and evidence of increased activity of coagulation factor VIII in patients with KS has been documented [6]. Also, the underlying hypoandrogenism causes increased plasma levels of plasminogen activator inhibitor-1 (PAI-1), which may trigger the formation of thrombi in patients with KS [7].

Other suggested hypotheses include the presence of comorbid conditions that could increase the risk of thrombosis in KS patients. These conditions include, but are not limited to, obesity, metabolic syndrome, diabetes mellitus, and systemic lupus erythematosus [8]. All of these probable comorbid conditions that could increase the risk of thrombosis were comfortably ruled out in our case.

This report has limitations. Levels of protein C, protein S, antithrombin III, and factor VIII were not assessed, as these tests were not routinely available at the treating center at the time of evaluation. It may have provided additional mechanistic insight into the hypercoagulable state. However, commonly screened inherited and acquired thrombophilic conditions were excluded.

Case reports of thrombophilia in KS patients with coexisting congenital hypercoagulable states, including methylenetetrahydrofolate reductase gene mutation [9,10], prothrombin (G20210A) mutations, and factor V Leiden mutations [11,12], are available in medical literature. In contrast, no such mutations were identified in the present patient, supporting Klinefelter syndrome as a contributing risk condition in the absence of other identifiable hereditary thrombophilia.

## Conclusions

This case highlights the rare but significant association between Klinefelter syndrome (KS) and thrombophilia. The diagnosis of chronic deep vein thrombosis (DVT) in our patient underscores the need for heightened clinical awareness of hypercoagulable states in individuals with KS, even in the absence of traditional risk factors for thrombosis. The comprehensive diagnostic approach, including hormonal assays and genetic studies, was crucial in supporting KS as an important contributing risk condition in the absence of other identifiable thrombophilic factors. Prompt anticoagulation therapy and patient education on the long-term implications of KS and its association with thrombophilia facilitated effective management and recovery. This case underscores the importance of considering KS in the differential diagnosis of unexplained thrombotic events and emphasizes the need for further research to elucidate the pathophysiological mechanisms linking KS and thrombophilia.
